# Stimuli-Responsive Nanoplatform-Assisted Photodynamic Therapy Against Bacterial Infections

**DOI:** 10.3389/fmed.2021.729300

**Published:** 2021-09-13

**Authors:** You Zhou, Wenmin Deng, Mulan Mo, Dexu Luo, Houhe Liu, Yuan Jiang, Wenjie Chen, Chuanshan Xu

**Affiliations:** ^1^Key Laboratory of Molecular Target & Clinical Pharmacology and the State & National Medical Products Administration Key Laboratory of Respiratory Disease, School of Pharmaceutical Sciences, The Fifth Affiliated Hospital, Guangzhou Medical University, Guangzhou, China; ^2^Department of Clinical Pharmacy, The People's Hospital of Dianbai District, Maoming, China; ^3^Department of Rehabilitation Medicine, The First Affiliated Hospital of Chengdu Medical College, Chengdu, China; ^4^State Key Laboratory of Respiratory Disease, Guangdong-Hongkong-Macao Joint Laboratory of Respiratory Infectious Disease, Guangzhou, China; ^5^Sydney Vital Translational Cancer Research Centre, Sydney, NSW, Australia

**Keywords:** bacterial infections, photodynamic therapy, stimuli-responsive, nanoplatforms, drug delivery systems

## Abstract

Bacterial infections are common diseases causing tremendous deaths in clinical settings. It has been a big challenge to human beings because of the antibiotics abuse and the newly emerging microbes. Photodynamic therapy (PDT) is a reactive oxygen species-based therapeutic technique through light-activated photosensitizer (PS). Recent studies have highlighted the potential of PDT as an alternative method of antibacterial treatment for its broad applicability and high efficiency. However, there are some shortcomings due to the low selectivity and specificity of PS. Growing evidence has shown that drug delivery nanoplatforms have unique advantages in enhancing therapeutic efficacy of drugs. Particularly, stimuli-responsive nanoplatforms, as a promising delivery system, provide great opportunities for the effective delivery of PS. In the present mini-review, we briefly introduced the unique microenvironment in bacterial infection tissues and the application of PDT on bacterial infections. Then we review the stimuli-responsive nanoplatforms (including pH-, enzymes-, redox-, magnetic-, and electric-) used in PDT against bacterial infections. Lastly, some perspectives have also been proposed to further promote the future developments of antibacterial PDT.

## Introduction

According to epidemiological reports, infections are a dominant contributor to the global disease burden. The mortality rates of bacterial infections are very high especially in developing countries, where medical resources such as vaccines and anti-infection therapeutics are less accessible (1). The use of antibiotics is a great milestone in fighting against bacterial infections. There are more than 38 antibiotics in clinical setting and 45 antibiotics have been undergoing clinical trials up to 2019. These antibiotics are developed to kill or inhibit the bacteria contagion through different mechanisms, including damage to the cell walls of bacteria, increase of the cell membrane permeability, and inhibition of the nucleic acid or protein synthesis (2). Due to antibiotics abuse, more and more bacteria have evolved the antibiotic resistance, which leads to the ineffectiveness of antibacterial therapy (3). Increasing antibiotic resistance among pathogenic bacteria is one of the most challenging issues in the present medical field; It is estimated that around 0.7 million people die every year, and it is predicted that maybe 10 million people every year will die from drug-resistant bacterial infections by 2050 (4). Thus, it is highly imperative to understand the underlying mechanisms of drug resistant infections and overcome them. In general, resistance to antibiotics occurs mainly through drug inactivation, composition and permeability modification, drug efflux, and acquired genetic resistance (5). There are increasing evidences revealing that many infections are caused by polybacteria, either in terms of origins or in manifestation. This may lead to limiting the therapeutic efficacy of a single antibiotic (6). To overcome the shortcomings of antibiotics, photodynamic therapy (PDT) has been developed as a promising alternative to treat bacterial infections with broad-spectrum and multitarget features (7, 8).

Photodynamic therapy efficacies rely on efficient delivery of photosensitizers (PSs). Stimuli-responsive nanoplatforms are a smart and promising delivery system that respond to endogenous stimuli (changes in pH, enzyme concentration, and redox gradients) or exogenous stimuli (magnetic field, ultrasound intensity, light, temperature, and electric pulses). These on-demand properties render the control of drug release in spatial–temporal and dosage-dependent manner (9). In this mini-review, we briefly introduced the unique microenvironment in bacterial infection tissues and the application of PDT on bacterial infections. Then we focus on the progress of stimuli-responsive nanoplatform-assisted antibacterial PDT. The potential opportunities and challenges will be also outlook to boost the developments of antibacterial PDT.

## Unique Microenvironment in Bacterial Infection Tissues

The unique microenvironment in bacterial infection tissues provides a prerequisite for designing stimuli-responsive nanoplatforms (10). The anaerobic fermentation, acidogenic/acid-tolerant bacteria metabolism, and local accumulation of organic acids such as lactic and acetic acids create an acidic microenvironment (pH 4.5–6.5) in infectious sites (11–14). Additionally, bacteria can secrete several protein virulence factors including lipases, esterases, proteases, hyaluronidases, alpha toxins, and chemotactic factors to protect themselves (15–17). These enzymes are highly expressed in Gram-positive bacteria, and they are likely to help bacteria to obtain energy, promote spread, escape from immune detection, etc. (18–20). Metalloproteinase 9 (MMP9) expressed in local infection acts as a synergistic virulence factor (21, 22). At the same time, surrounding tissues increase the detoxication components synthesis and induce the activation of defense response. For instance, an increase of glutathione (GSH) in the epithelial lining fluid was found in the *Pseudomonas aeruginosa* (*P. aeruginosa*) infection (23). Among the interaction between bacteria and hosts, a complicated inflammatory response is activated to combat infections (24–26). Interestingly, the excessive production of reactive oxygen species (ROS) by activated immune cells plays an essential role in the host immune defenses against pathogens, indicating a participation of oxidative stress in the pathology of bacterial infections (27).

### Application of PDT on Bacterial Infections

Photodynamic therapy is a clinically approved technique and mainly applied in treating cancerous and non-cancer diseases on the basis of ROS that generates from light-activated PS. Recently, PDT has also been employed to eliminate pathogens for treating the bacterial infections because it is less affected by the known antibiotic-resistance pathway (28). The successful applications of PDT are dependent on three essential factors, including light sources, PSs, and oxygen. When a PS is irradiated by light with a specific wavelength, it can be excited from the ground state to a triplet state. Then the excited PS can transfer electrons to molecular oxygen to generate superoxide anion and hydroxyl radicals, and hydrogen peroxide subsequently (Type I reaction). In another pathway, the excited PS transmits energy to ground triplet state oxygen to generate excited singlet oxygen and finally stimulates the bursting production of ROS (Type II reaction) (29). ROS in PDT induce a lethal oxidative damage to biological macromolecules like membrane lipids and nucleic acids (30). Beyond the direct killing of pathogenic bacteria through structural disruption, studies found that PDT treatment can induce inactivation of physiologic function-relative protein in bacteria (31, 32). Furthermore, bacterial virulence factors can also be inactivated under PDT treatment (33). Limited by a short lifespan and diffusion distance of ROS, the cellular targets of ROS are mainly dependent on the cellular localization of the PS (34, 35). Innate immunity in host may also affect the therapy effect since the attraction and accumulation of neutrophils into the infection regions were required for PDT-mediated bacteria killing and infection clearance (36). The multitargets of ROS oxidation to biomacromolecules make bacteria hard to develop a resistance to PDT, but studies indicate that the efficiency of PDT could be affected by bacteria strains, genetic background, and its surrounding microenvironment (37–39). Mechanism underlying such a phenomenon is quite complex. Nevertheless, the correlations between the responses of different strains to PDT and the antioxidative systems, cell membrane contents, biofilm production ability, quorum sensing signaling systems have been previously reviewed (40–43).

The wide spectrum and credible effects have already promoted the clinical trials of PDT in antibacterial treatment (Supplementary Table 1). But there are still some shortcomings attracting an attention, such as the water-insolubility of PS, insufficient uptakes of PS by pathogenic bacteria, the oxygen shortage in infection lesions, and the phototoxicity-induced side-effects (44). New PS and drug delivery systems are under development, in which stimuli-responsive nanoplatforms show an important role in promoting ROS production and enhancing antibacterial PDT efficacy (8).

### Stimuli-Responsive Nanoplatform-Assisted PDT on Bacterial Infections

The PS delivery is developed to improve efficacy of the conventional antibacterial PDT approaches. Owing to the advantages in both pharmacokinetics and pharmacodynamics, stimuli-responsive nanoplatforms have been applied to overcome the issues of poor delivery performance (45). In the following section, we will review stimuli-responsive Nanoplatform-Assisted PDT in fighting against bacterial infections. These specific stimuli include pH, enzymes, redox gradients, magnetic and electric field, and will be presented systematically below.

#### pH-Responsive Nanoplatform-Assisted PDT in Bacterial Infections

Chemical reactions, protonation, or degradation of administrated compounds can occur under acidic circumstances. Therefore, the pH-responsive strategy has been applied in antibacterial PDT. Polyacrylic acid (PAA) is often used in the delivery of PS for its pH responsive property. Hao et al. synthesized zeolitic imidazolate framework-8 (ZIF-8) for the local delivery of ammonium methylbenzene blue. Then PAA is incorporated for pH responsiveness and higher drug loading capacity. This nanoplatform had long blood circulation in physiological environment and pH responsive drug release in bacterial infection site. The *in vitro* and *in vivo* experiments showed better therapeutic efficacy than PS treatment alone (46). Similar to the above mentioned platform, Perni et al. constructed a silica–toluidine blue O (TBO) nanoconjugate by the formation of amide bonds between silica nanoparticles and TBO. The controlled delivery of TBO released from the conjugates because of the amide bond cleavage in bacterial infection tissues. In this research, TBO shows an enhanced photosensitive activity in eliminating methicillin-resistant *Staphylococcus aureus* (MRSA), *Staphylococcus epidermidi*s (*S. epidermidis*), and *Escherichia coli* (*E. coli*) (47). Protonation in low pH environment often results in a charge change, which is conducive to the accumulation of PS in bacteria surface with negative charge. Thus, Wang et al. used chlorin e6 (Ce6)-linked supramolecule to develop a self-assembled micelle to treat bacterial infections. The negatively-charged micelles could change to positive charge in bacterial infection sites, subsequently adhering to bacteria membranes. This micelle significantly enhanced the inhibition effect of Ce6-based PDT on a variety of bacteria including MRAS, and showed a great anti-infection activity in the subcutaneous infection model (48). The strategy was also implemented in Ce6 loaded SiO_2_-polymer nanoparticles (named SiO_2_-P_Ce6−IL_). Ce6 COO- and 1-vinyl imidazole with dodecyl were assembled by anion exchange reaction, and SiO_2_ nanoparticles were introduced to control the density of Ce6-IL polymers. With the protonation of Ce6 in bacterial infection sites, the charge of SiO_2_-P_Ce6−IL_ was inverted from negative to positive. And the acquired positive charge of polymer led to an interaction of SiO_2_-P_IL_+ with negative charge extracellular polymeric substances, which induced a rapid release of Ce6 from nanoparticles and dramatically improved the PDT efficacy against MRSA biofilm infection (49). Aggregation-induced emission (AIE) PSs exhibit potential application prospect in the PDT because of their aggregation-enhanced ROS production (50). Bibo et al. fabricated an AIE PS loaded in the zwitterionic polyurethane nanomicelles. The AIE PS aggregated around bacteria when zwitterionic moiety acquired positive charge by acid protonation, thus the nanomicelle achieved a superior antibacterial activity (51). In addition to the delivery of PS, low pH also offers possibilities for overcoming the limitations of low oxygen level. Since manganese dioxide (MnO_2_) can produce oxygen by its catalytic activity at low pH and high H_2_O_2_ in the infected sites, Deng et al. synthesized a multicomponent nanoparticle by coencapsulating ultrasmall-sized Hf (IV)-porphyrin Metal-Organic Framework (MOF) and MnO_2_ in human serum albumin. In this nanoplatform, the production rate of ROS was much higher than porphyrin-based MOF treatment alone by *in situ* O_2_ generation. With the alleviated hypoxia, it realizes great therapeutic outcomes in *S aureus*–infected models (52). Meanwhile, the magnetic resonance signal of Mn^2+^ provides the detection of bacteria, which is favorable to build up the theranostic platform (53, 54).

#### Enzymes-Responsive Nanoplatform-Assisted PDT in Bacterial Infections

The specific high expression and selective catalytic activity endow enzymes with an excellent trigger for controlled delivery of drugs. Based on the cleavage of ester linkage by lipase, more than one team have reported lipase-responsive nanoplatforms for anti-bacterial PDT (55–57). For example, nanoliposomes were used to deliver PS pheophorbide A. Erythromycin-loaded liposomes were coated with pullulan-pheophorbide A conjugates. Once the nanoplatform reached the infection site, pheophorbide A and erythromycin release were triggered by the lipase-dependent cleavage of ester linkage in lipid as well as the one between pullulan and pheophorbide A. This formulation fulfilled the synergistic therapy in skin infection by PS and erythromycin codelivery (55). In another study, pheophorbide A was conjugated with DSPE-PEG to form the nanoliposome, and the photoactivity of pheophorbide A quenched in spherical shape was gradually recovered by the cleavage of ester linkage by *P. acnes* lipases in infection foci, and the formation of nanoliposome enhanced the skin penetration of pheophorbide A (56). Songhee et al. generated a hypocrellin A-loaded methoxy poly (ethylene glycol)-block-poly(ε-caprolactone) polymer micelle. This lipase-sensitive micelle not only overcomes the aggregation and low water solubility of hypocrellin A *in vivo*, but also enhances the efficiency of PDT in a MRSA-induced acute peritonitis model (57). For developing MMP-responsive nanoplatform, MMP9 sensitive peptide (YGRKKKRRQRRR-GPLGVRG-EEEEEE) was conjugated with Ce6 to construct a polypeptide nanoparticle. Negatively charged surface by EEEEEE peptide shell was removed by overexpressed MMP9 in the keratitis microenvironment. Subsequently, the exposed cationic peptides helped the nanoparticles to penetrate and accumulate in biofilms as well as bind to Gram-negative bacteria, thereby improving the antibacterial PDT efficacy (58). Hyaluronidase secreted by MRSA was also applied as an endogenous stimulus in antibacterial PDT. Yuwen et al. prepared a MoS2@HA-Ce6 nanosheet. MoS2 nanosheets served as a fluorescence quencher, and hyaluronic acid conjugated with Ce6 (HA–Ce6) was assembled on the surface of MoS2 nanosheets. These nanosheets could restore the photodynamic activity of Ce6 after the hyaluronic acid shell degraded by hyaluronidase, and *in vivo* study shows an excellent MRSA eradication effect (59).

#### Redox-Responsive Nanoplatform-Assisted PDT in Bacterial Infections

The high levels of GSH and/or H_2_O_2_ in bacteria-infected lesions provides alternatives for the design of redox-responsive systems. Michael et al. conjugated porphyrin to hyperbranched polyglycerol nanoparticles with disulfide linker with GSH-responsive property. Experiments *in vitro* showed that disulfide linker in conjugates could significantly improve the phototoxicity of porphyrin against *S. aureus*, despite the lack of additional targeting groups (60). Mao et al. reported a metabolic labeling strategy for precise delivery of AIE PS. They used MIL-100 (Fe), a MOF composed of iron (III) metal centers and trimesic acid ligand nanoparticles, as the carrier for 3-azido-d-alanine delivery firstly. MIL-100 (Fe) would be dissociated as a result of coordination breaking between trimesic acid and iron (III). In this way, the specific release of the encapsulated 3-azido-d-alanine can be achieved. When MIL-100 (Fe) accumulated and degraded within the infection environment with high levels of H_2_O_2_, 3-azido-d-alanine was released and selectively integrated into the cell walls of bacteria. Then the dibenzocyclooctyne-modified AIE PS nanoparticles can subsequently react with the 3-azido-d-alanine labeled bacteria. Through this modification, the implementation of PDT in the infected tissue can significantly reduce bacteria growth (61).

#### External Stimuli-Responsive Nanoplatforms-Assisted PDT in Bacterial Infections

Different from the internal stimuli, external stimuli can control the drug release more precisely, and external stimuli can be controlled accurately either by the local or the intensity to meet the treatment requirements (62, 63). For example, magnetic field has been used in the targeted delivery of magnetic materials. Sun et al. developed a Ce6-and C6-loaded Fe_3_O_4_-silane core-shell nanoparticle to fight against periodontal biofilms growing in dentin disks. Under the magnetically driven force, this nanoparticle could increase the penetration of the PS into biofilms. The results demonstrated that the magnetic nanoparticle had a strong antibiofilm activity with excellent biocompatibility, real-time monitoring, and magnetically-targeting capacities (64). Several other magnetic nanoparticles have constructed on the basis of the iron or Fe_3_O_4_ magnetic properties to deliver PS. They enhanced bacterial killing significantly by achieving targeting and combination therapy (65–68). In addition to the magnetic field, the electric field has also served as a novel switch for controlled delivery in PDT on infection. Steven et al. designed an electric-responsive hydrogel carrying PS to treat wound infections. The hydrogel was composed of polyelectrolyte poly (methyl vinyl ether-co-maleic acid) (PMVE-co-MA), which can regulate the ionic conductivities of the hydrogel by PMVE/MA concentration ratio. The hydrogel acquired a rapid release of excess PS in a PMVE/MA ratio-dependent manner upon electric stimulation. Thus, this electric responsive hydrogel is a potential option in antibacterial PDT at an open wound (69).

### Summary and Outlook

**Figure 1:**
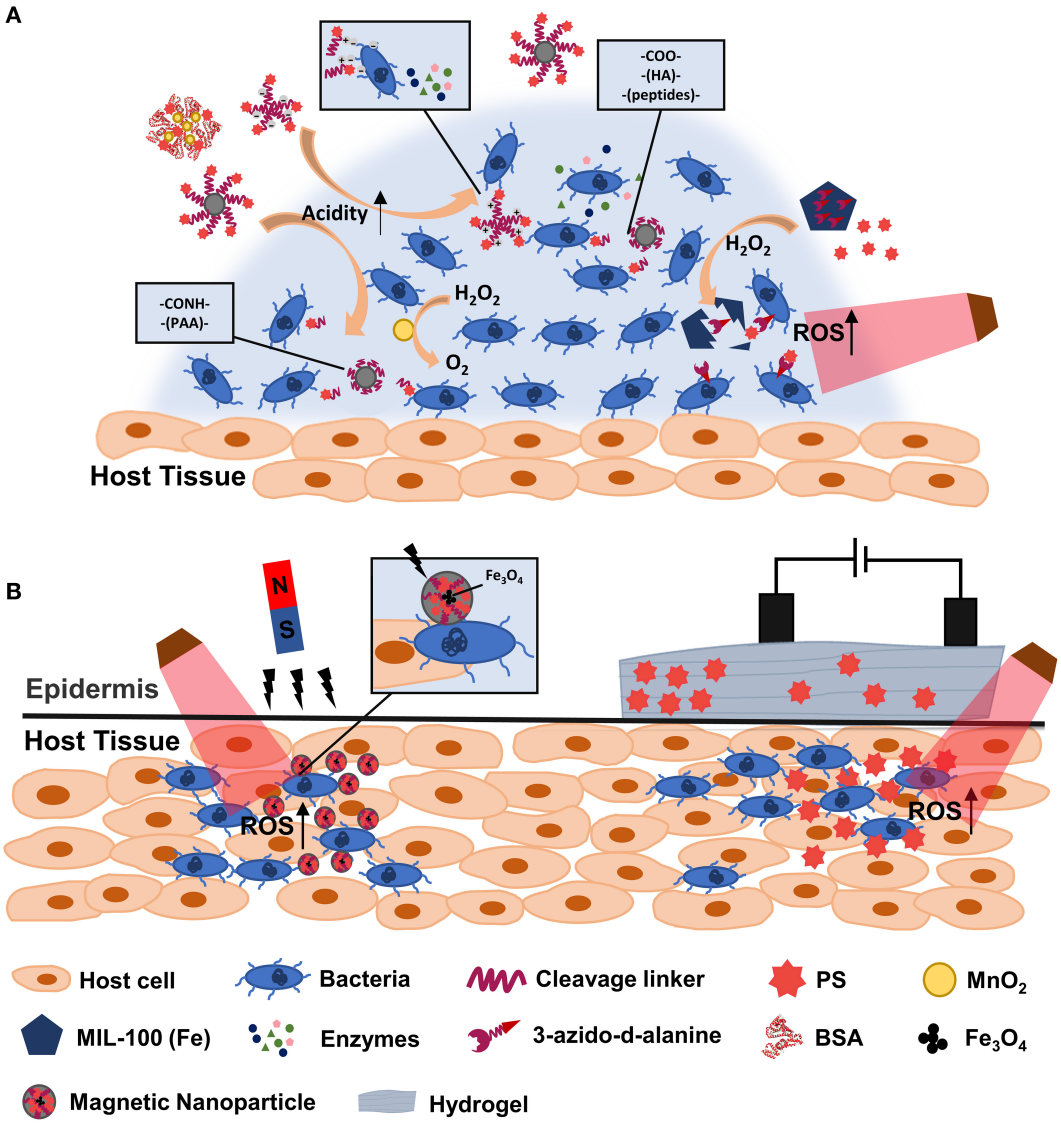
Scheme illustration of stimuli-responsive Nanoplatforms-Assisted PDT in fighting against bacterial infections. Internal **(A)** and external **(B)** stimuli-responsive platform assisted PDT are showed. PAA, polyacrylic acid; HA, hyaluronic acid; BSA, bovine serum albumin.

Stimuli-responsive nanoplatforms have unique advantages in spatial and temporal manipulations of drug release and elongation of blood retention, which provide a promising strategy for drug delivery (70). Currently, regarding unique microenvironment of bacterial infections, various responsive strategies have been widely developed on the basis of pH, enzymes, and redox gradients to improve the efficiency of antibacterial PDT. In addition, external factors such as magnetic and electric field were also applied as trigger sources (Figure 1). Compared with already recent developments, stimuli-responsive platforms can not only improve the solubility of PS, but also confer other advantages (Table 1). On one hand, stimuli-responsive platforms triggered by the unique microenvironment in bacterial-infected tissues confer the selective drug release. On the other hand, these nanoplatforms can provide targeting ability for PS, for example, by stimuli-responsive charge converting and magnetically-driven force. Therefore, stimuli-responsive platforms avoided potential side-effects and enhanced the PDT eradication of bacterial infections. What is more, the codelivery of stimuli-responsive catalytic activity components like manganese dioxide (MnO_2_) can lead to an *in situ* oxygen generation in bacterial infection sites for hypoxia alleviation (52). Lastly, stimuli-responsive nanoplatforms with excellent loading performance exhibit broad prospects for synergetic therapies (55). These properties make stimuli-responsive nanoplatforms a great option in assisting PDT for fighting against bacterial infections. Among them, the external stimuli-responsive platforms may be superior to those responsive to internal stimuli since they can be designed to artificially control the drug release more easily. Besides, a dual-responsive nanoplatform with more smart features may have greater potential to further enhance the antibacterial PDT efficacy.

**Table 1:** Summary of stimuli-responsive Nanoplatforms-Assisted PDT in anti-bacterial.

**Source of respond**	**Responsive method**	**Bacteria species**	**PS**	**Vehicle**	**Advantages**	**References**
pH	Proton; Amide bond break; PAA; MnO_2_ catalysis	MRSA; *S. epidermidis*; *E. coil*; *S. aureus*	Ce6; TBO; DHTPY; ICG; Porphyrin; MB	Micelle; Nanoparticle; MOF; ZIF-8; Nanosheet;	Enhance bacterial affinity; Control delivery; Enhance biocompatibility; Prolong circulation; Targeting property; High ROS generation; Biofilm penetration; Hypoxia alleviation; Bioimaging functionality; Synergistic effect	(46–54)
Enzyme	Ester linkage; Hyaluronic acid; MMP-9-sensitive peptides	*P. acnes*; MRSA; *P. aeruginosa*;	Pheo A; HA; Ce6;	Liposome; Micelle; Nanosheet; Nanoparticle;	Prevent PS aggregation; Enhance water solubility; High selectivity and penetration property; Enhance retention time	(55–59)
Redox	MIL-100 (Fe); Disulfide bond	MRSA	TPETM; Porphyrin	Nanoparticle	Precise detection and therapy; Multivalent targeting	(60, 61)
Magnetic	Fe_3_O_4_; Fe	*S. sanguinis*; *P. gingivalis*; *F. nucleatum*; *Salmonella* DT104; MRSA; VRE; *E. faecalis*; *S. aureus*; *B. cereus*; MSSA; *E. coli*; *S. typhimurium*	Ce6/C6; MB; MgPc; t-PtCP	Nanoparticle	Good biocompatibility; Real-time monitoring; Magnetically-targeting; Multimodel treatment; Improve ROS generation	(64–68)
Electric	Hydrogel lysis	MRSA	TMP; MB	Hydrogel	Rapid release	(69)

*MRSA, Methicillin-resistant Staphylococcus aureus; S. epidermidis, Staphylococcus epidermidis; E. coil, Escherichia coli; S. aureus, Staphylococcus aureus; P. acnes, Propionibacterium acnes; P. aeruginosa, Pseudomonas aeruginosa; S. sanguinis, Streptococcus sanguinis; P. gingivalis, Porphyromonas gingivalis; F. nucleatum, Fusobacterium nucleatum; VRE, (VAN)-resistant Enterococcus; E. faecalis, Enterococcus faecalis; B. cereus, Bacillus cereus; MSSA, Methicillin-sensitive Staphylococcus aureus; S. typhimurium, Salmonella typhimurium. Ce6, Chlorin e6; TBO, Toluidine blue O; DHTPY, Pyridinium, 1-(2,3-dihydroxypropyl)-4-[2-[4-(diphenylamino)phenyl]ethenyl]; ICG, Indocyanine green; MB, Methylbenzene blue; Pheo A, Pheophorbide A; HA, Hypocrellin A; TPETM, 2-(1-(5-(4-(1,2,2-tris(4-methoxyphenyl)vinyl)phenyl)th-iophen-2-yl)ethylidene)malononitrile; C6, Coumarin 6; MgPc, Mg (II) or Al (III) phthalocyanine; t-PtCP, [5,15-bisphenyl-10,20-bis(4-methoxy-carbonylphenyl)-porphyrin] platinum; TMP, meso-Tetra (N-methyl-4-pyridyl) porphine tetra tosylate*.

Recent evidences have shown that the stimuli-responsive nanoplatform-assisted PDT is a promising way to combat bacterial infections *in vitro* and *in vivo*. However, a stimuli-responsive property often means more tedious preparation and complicated characterization. Additionally, there are heterogenicity among bacterial species and patient population in the application process, these diversities may make the stimuli-responsive elements ineffective and ultimately affect the drug-releasing and PDT efficacy. While this problem can be settled by personalized-medicine approaches, it needs a complex diagnostic approach (13). Currently, the encouraging stimuli-responsive Nanoplatforms-Assisted PDT in bacterial infections are all reported by *in vitro* and *in vivo* experimental studies, but no clinical trials are undergoing so far. The curative effect of these nanoplatforms needs to be demonstrated by more extensive clinical data. Furthermore, there are challenges such as uncertainty of the *in vivo* fate of nanoplatforms, limiting the development of stimuli-responsive nanoplatforms. Therefore, further addressing the above shortcomings should be an important task for translating stimuli-responsive nanoplatform-assisted PDT to clinical antibacterial infections.

## Author Contributions

YZ, CX, and WC contributed to the conception and structure of this review. YZ prepared the manuscript. WD did the literature search and wrote the introduction. CX and WC reviewed and revised the manuscript. MM, DL, HL, and YJ provided written comments. All authors contributed to the article and approved the submitted version.

## Funding

This work was supported by the grants of High-level University Construction Fund of Guangdong Province (Nos. 06-410-2106154, 06-410-2106153, and 06-410-2107229) and the fund of Guangdong-Hongkong-Macao Joint Laboratory of Respiratory Infectious Disease for WC.

## Conflict of Interest

The authors declare that the research was conducted in the absence of any commercial or financial relationships that could be construed as a potential conflict of interest.

## Publisher's Note

All claims expressed in this article are solely those of the authors and do not necessarily represent those of their affiliated organizations, or those of the publisher, the editors and the reviewers. Any product that may be evaluated in this article, or claim that may be made by its manufacturer, is not guaranteed or endorsed by the publisher.

## References

[1] GBD 2017 Mortality Collaborators. Global, regional, and national age-sex-specific mortality for 282 causes of death in 195 countries and territories, 1980-2017: a systematic analysis for the Global Burden of Disease Study 2017. Lancet. (2018) 392:1736–88. 10.1016/S0140-6736(18)31891-9 30496103 PMC6227606

[2] Hutchings MI Truman AW Wilkinson B. Antibiotics: past, present and future. Curr Opin MicrobiolI. (2019) 51:72–80. 10.1016/j.mib.2019.10.008 31733401

[3] Morehead MS Scarbrough C. Emergence of Global Antibiotic Resistance. Prim Care. (2018) 45:467–84. 10.1016/j.pop.2018.05.006 30115335

[4] O'Neill J. Tackling drug-resistant infections globally: final report and recommendations. Rev Antimicrobal Resist. (2016). Available online at: https://amr-review.org

[5] Munita JM Arias CA. Mechanisms of antibiotic resistance. Microbiol Spectr. (2016) 4:10. 10.1128/microbiolspec.VMBF-0016-2015 27227291 PMC4888801

[6] Tay WH Chong KK Kline KA. Polymicrobial-host interactions during infection. J Mol Biol. (2016) 428:3355–71. 10.1016/j.jmb.2016.05.006 27170548

[7] Dehghan EM Bozorgmehr A Hajjari SN Sadat SA Malekshahi ZV Sadeghizadeh M. Review of new insights into antimicrobial agents. Cell Mol Biol. (2017) 63:40–8. 10.14715/cmb/2017.63.2.6 28364794

[8] Jia Q Song Q Li P Huang W. Rejuvenated photodynamic therapy for bacterial infections. Adv Healthc Mater. (2019) 8:e1900608. 10.1002/adhm.201900608 31240867

[9] Li L Yang WW Xu DG. Stimuli-responsive nanoscale drug delivery systems for cancer therapy. J Drug Target. (2019) 27:423–33. 10.1080/1061186X.2018.1519029 30173577

[10] Wells CM Harris M Choi L Murali VP Guerra FD Jennings JA. Stimuli-responsive drug release from smart polymers. J Funct Biomater. (2019) 10:34. 10.3390/jfb10030034 31370252 PMC6787590

[11] Koo H Falsetta ML Klein MI. The exopolysaccharide matrix: a virulence determinant of cariogenic biofilm. J Dent Res. (2013) 92:1065–73. 10.1177/0022034513504218 24045647 PMC3834652

[12] Liu Y Ren Z Hwang G Koo H. Therapeutic strategies targeting cariogenic biofilm microenvironment. Adv Dent Res. (2018) 29:86–92. 10.1177/0022034517736497 29355421 PMC5784482

[13] Benoit DS Koo H. Targeted, triggered drug delivery to tumor and biofilm microenvironments. Nanomedicine. (2016) 11:873–9. 10.2217/nnm-2016-0014 26987892

[14] Guo L McLean JS Lux R He X Shi W. The well-coordinated linkage between acidogenicity and aciduricity *via* insoluble glucans on the surface of *Streptococcus mutans*. Sci Rep. (2015) 5:18015. 10.1038/srep18015 26657939 PMC4675080

[15] Nisar S Kirkpatrick LD Shupp JW. Bacterial virulence factors and their contribution to pathophysiology after thermal injury. Surg Infect. (2021) 22:69–76. 10.1089/sur.2020.188 32735479

[16] Knor T. The pathogenesis of acne. Acta Dermatovenerol Croat. (2005) 13:44–9. 10.1016/0021-9681(85)90103-1 15788147

[17] Holland C Mak TN Zimny-Arndt U Schmid M Meyer TF Jungblut PR. Proteomic identification of secreted proteins of Propionibacterium acnes. BMC Microbiol. (2010) 10:230. 10.1186/1471-2180-10-230 20799957 PMC3224659

[18] Starr CR Engleberg NC. Role of hyaluronidase in subcutaneous spread and growth of group A streptococcus. Infect Immun. (2006) 74:40–8. 10.1128/IAI.74.1.40-48.2006 16368955 PMC1346594

[19] Escosura-Muniz A Ivanova K Tzanov T. Electrical evaluation of bacterial virulence factors using nanopores. ACS Appl Mater Interfaces. (2019) 11:13140–6. 10.1021/acsami.9b02382 30888786

[20] Vornhagen J Quach P Boldenow E Merillat S Whidbey C Ngo LY. Bacterial hyaluronidase promotes ascending GBS infection and preterm birth. mBio. (2016) 7:16. 10.1128/mBio.00781-16 27353757 PMC4937215

[21] Miyoshi S Shinoda S. Microbial metalloproteases and pathogenesis. Microbes Infect. (2000) 2:91–8. 10.1016/S1286-4579(00)00280-X 10717546

[22] Jamerson EC Elhusseiny AM ElSheikh RH Eleiwa TK El SY. Role of matrix metalloproteinase 9 in ocular surface disorders. Eye Contact Lens. (2020) 46(Suppl.2):S57–63. 10.1097/ICL.0000000000000668 32068662

[23] Day BJ van Heeckeren AM Min E Velsor LW. Role for cystic fibrosis transmembrane conductance regulator protein in a glutathione response to bronchopulmonary pseudomonas infection. Infect Immun. (2004) 72:2045–51. 10.1128/IAI.72.4.2045-2051.2004 15039325 PMC375208

[24] Zhang L Wang CC. Inflammatory response of macrophages in infection. Hepatobiliary Pancreat Dis Int. (2014) 13:138–52. 10.1016/S1499-3872(14)60024-2 24686541

[25] Jenne CN Kubes P. Platelets in inflammation and infection. Plateles. (2015) 26:286–92. 10.3109/09537104.2015.1010441 25806786

[26] Syed S Viazmina L Mager R Meri S Haapasalo K. Streptococci and the complement system: interplay during infection, inflammation and autoimmunity. FEBS Lett. (2020) 594:2570–85. 10.1002/1873-3468.13872 32594520

[27] Novaes RD Teixeira AL de Miranda AS. Oxidative stress in microbial diseases: pathogen, host, and therapeutics. Oxid Med Cell Longev. (2019) 2019:8159562. 10.1155/2019/8159562 30774746 PMC6350582

[28] Shi X Zhang CY Gao J Wang Z. Recent advances in photodynamic therapy for cancer and infectious diseases. Wiley Interdiscip Rev Nanomed Nanobiotechnol. (2019) 11:e1560. 10.1002/wnan.1560 31058443 PMC6697192

[29] Foote CS. Definition of type I and type II photosensitized oxidation. Photochem Photobiol. (1991) 54:659. 10.1111/j.1751-1097.1991.tb02071.x 1798741

[30] Almeida A Faustino MA Tome JP. Photodynamic inactivation of bacteria: finding the effective targets. Future Med Chem. (2015) 7:1221–4. 10.4155/fmc.15.59 26144260

[31] Dosselli R Millioni R Puricelli L Tessari P Arrigoni G Franchin C. Molecular targets of antimicrobial photodynamic therapy identified by a proteomic approach. J Proteomics. (2012) 77:329–43. 10.1016/j.jprot.2012.09.007 23000218

[32] Chang KC Cheng YY Lai MJ Hu A. Identification of carbonylated proteins in a bactericidal process induced by curcumin with blue light irradiation on imipenem-resistant *Acinetobacter baumannii*. Rapid Commun Mass Spectrom. (2020) 34 Suppl 1:e8548. 10.1002/rcm.8548 31397940

[33] Braham P Herron C Street C Darveau R. Antimicrobial photodynamic therapy may promote periodontal healing through multiple mechanisms. J Periodontol. (2009) 80:1790–8. 10.1902/jop.2009.090214 19905948

[34] Baier J Maier M Engl R Landthaler M Baumler W. Time-resolved investigations of singlet oxygen luminescence in water, in phosphatidylcholine, and in aqueous suspensions of phosphatidylcholine or HT29 cells. J Phys Chem B. (2005) 109:3041–6. 10.1021/jp0455531 16851318

[35] Maisch T Baier J Franz B Maier M Landthaler M Szeimies RM. The role of singlet oxygen and oxygen concentration in photodynamic inactivation of bacteria. Proc Natl Acad Sci USA. (2007) 104:7223–8. 10.1073/pnas.0611328104 17431036 PMC1851884

[36] Tanaka M Mroz P Dai T Huang L Morimoto Y Kinoshita M. Photodynamic therapy can induce a protective innate immune response against murine bacterial arthritis via neutrophil accumulation. PLoS ONE. (2012) 7:e39823. 10.1371/journal.pone.0039823 22761911 PMC3383702

[37] Nakonieczna J Michta E Rybicka M Grinholc M Gwizdek-Wisniewska A Bielawski KP. Superoxide dismutase is upregulated in *Staphylococcus aureus* following protoporphyrin-mediated photodynamic inactivation and does not directly influence the response to photodynamic treatment. BMC Microbiol. (2010) 10:323. 10.1186/1471-2180-10-323 21167031 PMC3022707

[38] Rapacka-Zdonczyk A Larsen AR Empel J Patel A Grinholc M. Association between susceptibility to photodynamic oxidation and the genetic background of *Staphylococcus aureus*. Eur J Clin Microbiol Infect Dis. (2014) 33:577–86. 10.1007/s10096-013-1987-5 24158686 PMC3953553

[39] Hu X Huang YY Wang Y Wang X Hamblin MR. Antimicrobial photodynamic therapy to control clinically relevant biofilm infections. Front Microbiol. (2018) 9:1299. 10.3389/fmicb.2018.01299 29997579 PMC6030385

[40] Grinholc M Szramka B Kurlenda J Graczyk A Bielawski KP. Bactericidal effect of photodynamic inactivation against methicillin-resistant and methicillin-susceptible *Staphylococcus aureus* is strain-dependent. J Photochem Photobiol B. (2008) 90:57–63. 10.1016/j.jphotobiol.2007.11.002 18093839

[41] Rapacka-Zdonczyk A Wozniak A Michalska K Pieranski M Ogonowska P Grinholc M. Factors determining the susceptibility of bacteria to antibacterial photodynamic inactivation. Front Med. (2021) 8:642609. 10.3389/fmed.2021.642609 34055830 PMC8149737

[42] Orlandi VT Bolognese F Martegani E Cantaluppi V Medana C Barbieri P. Response to photo-oxidative stress of *Pseudomonas aeruginosa* PAO1 mutants impaired in different functions. Microbiology. (2017) 163:1557–67. 10.1099/mic.0.000543 29022867

[43] Mahdizade-Ari M Pourhajibagher M Bahador A. Changes of microbial cell survival, metabolic activity, efflux capacity, and quorum sensing ability of *Aggregatibacter actinomycetemcomitans* due to antimicrobial photodynamic therapy-induced bystander effects. Photodiagnosis Photodyn Ther. (2019) 26:287–94. 10.1016/j.pdpdt.2019.04.021 31026616

[44] Qi M Chi M Sun X Xie X Weir MD Oates TW. Novel nanomaterial-based antibacterial photodynamic therapies to combat oral bacterial biofilms and infectious diseases. Int J Nanomedicine. (2019) 14:6937–56. 10.2147/IJN.S212807 31695368 PMC6718167

[45] Mura S Nicolas J Couvreur P. Stimuli-responsive nanocarriers for drug delivery. Nat Mater. (2013) 12:991–1003. 10.1038/nmat3776 24150417

[46] Chen H Yang J Sun L Zhang H Guo Y Qu J. Synergistic chemotherapy and photodynamic therapy of endophthalmitis mediated by zeolitic imidazolate framework-based drug delivery systems. Small. (2019) 15:e1903880. 10.1002/smll.201903880 31588682

[47] Perni S Drexler S Ruppel S Prokopovich P. Lethal photosensitisation of bacteria using silica-TBO nanoconjugates. Colloid Surface A. (2016) 510:293–9. 10.1016/j.colsurfa.2016.06.022

[48] Wang S Fang Y Zhang Z Jin Q Ji J. Bacterial infection microenvironment sensitive prodrug micelles with enhanced photodynamic activities for infection control. Colloid Interface Sci. (2021) 40:100354. 10.1016/j.colcom.2020.100354

[49] Wang C Chen P Qiao Y Kang Y Yan C Yu Z. pH responsive superporogen combined with PDT based on poly Ce6 ionic liquid grafted on SiO_2_ for combating MRSA biofilm infection. Theranostics. (2020) 10:4795–808. 10.7150/thno.42922 32308750 PMC7163436

[50] Bai H He W Chau J Zheng Z Kwok R Lam J. AIEgens for microbial detection and antimicrobial therapy. Biomaterials. (2021) 268:120598. 10.1016/j.biomaterials.2020.120598 33321291

[51] Ren B Li K Liu Z Liu G Wang H. White light-triggered zwitterionic polymer nanoparticles based on an AIE-active photosensitizer for photodynamic antimicrobial therapy. J Mater Chem B. (2020) 8:10754–63. 10.1039/D0TB02272A 33155608

[52] Deng Q Sun P Zhang L Liu Z Wang H Ren J. Porphyrin MOF dots-based, function -adaptive nanoplatform for enhanced penetration and photodynamic eradication of bacterial biofilms. Adv Funct Mater. (2019) 29:1903018. 10.1002/adfm.201903018

[53] Lu X Chen R Lv J Xu W Chen H Ma Z. High-resolution bimodal imaging and potent antibiotic/photodynamic synergistic therapy for osteomyelitis with a bacterial inflammation-specific versatile agent. Acta Biomater. (2019) 99:363–72. 10.1016/j.actbio.2019.08.043 31465882

[54] Xiu W Gan S Wen Q Qiu Q Dai S Dong H. Biofilm microenvironment-responsive nanotheranostics for dual-mode imaging and hypoxia-relief-enhanced photodynamic therapy of bacterial infections. Research. (2020) 2020:9426453. 10.34133/2020/9426453 32377640 PMC7128073

[55] Jeong S Lee J Im BN Park H Na K. Combined photodynamic and antibiotic therapy for skin disorder via lipase-sensitive liposomes with enhanced antimicrobial performance. Biomaterials. (2017) 141:243–50. 10.1016/j.biomaterials.2017.07.009 28697465

[56] Park H Lee J Jeong S Im BN Kim MK Yang SG. Lipase-sensitive transfersomes based on photosensitizer/polymerizable lipid conjugate for selective antimicrobial photodynamic therapy of acne. Adv Healthc Mater. (2016) 5:3139–47. 10.1002/adhm.201600815 27863184

[57] Guo LY Yan SZ Tao X Yang Q Li Q Wang TS. Evaluation of hypocrellin A-loaded lipase sensitive polymer micelles for intervening methicillin-resistant *Staphylococcus Aureus* antibiotic-resistant bacterial infection. Mater Sci Eng C Mater Biol Appl. (2020) 106:110230. 10.1016/j.msec.2019.110230 31753349

[58] Han H Gao Y Chai M Zhang X Liu S Huang Y. Biofilm microenvironment activated supramolecular nanoparticles for enhanced photodynamic therapy of bacterial keratitis. J Control Releas. (2020) 327:676–87. 10.1016/j.jconrel.2020.09.014 32920078

[59] Yuwen L Qiu Q Xiu W Yang K Li Y Xiao H. Hyaluronidase-responsive phototheranostic nanoagents for fluorescence imaging and photothermal/photodynamic therapy of methicillin-resistant Staphylococcus aureus infections. Biomater Sci. (2021) 9:4484–95. 10.1039/D1BM00406A 34002742

[60] Staegemann MH Grafe S Gitter B Achazi K Quaas E Haag R. Hyperbranched polyglycerol loaded with (zinc-)porphyrins: photosensitizer release under reductive and acidic conditions for improved photodynamic therapy. Biomacromolecules. (2018) 19:222–38. 10.1021/acs.biomac.7b01485 29232113

[61] Mao D Hu F Kenry Ji S Wu W Ding D. Metal-organic-framework-assisted *in vivo* bacterial metabolic labeling and precise antibacterial therapy. Adv Mater. (2018) 30:e1706831. 10.1002/adma.201706831 29504163

[62] Raza A Rasheed T Nabeel F Hayat U Bilal M Iqbal H. Endogenous and exogenous stimuli-responsive drug delivery systems for programmed site-specific release. Molecules. (2019) 24:1117. 10.3390/molecules24061117 30901827 PMC6470858

[63] Mi P. Stimuli-responsive nanocarriers for drug delivery, tumor imaging, therapy and theranostics. Theranostics. (2020) 10:4557–88. 10.7150/thno.38069 32292515 PMC7150471

[64] Sun X Wang L Lynch CD Sun X Li X Qi M. Nanoparticles having amphiphilic silane containingChlorin e6 with strong anti-biofilm activity against periodontitis-related pathogens. J Dent. (2019) 81:70–84. 10.1016/j.jdent.2018.12.011 30593855

[65] Dai X Fan Z Lu Y Ray PC. Multifunctional nanoplatforms for targeted multidrug-resistant-bacteria theranostic applications. ACS Appl Mater Interfaces. (2013) 5:11348–54. 10.1021/am403567k 24138085

[66] Idowu MA Xego S Arslanoglu Y Mark J Antunes E Nyokong T. Photophysicochemical behaviour and antimicrobial properties of monocarboxy Mg (II) and Al (III) phthalocyanine-magnetite conjugates. Spectrochim Acta A Mol Biomol Spectrosc. (2018) 193:407–14. 10.1016/j.saa.2017.12.052 29277071

[67] Choi KH Lee HJ Park BJ Wang KK Shin EP Park JC. Photosensitizer and vancomycin-conjugated novel multifunctional magnetic particles as photoinactivation agents for selective killing of pathogenic bacteria. Chem Commun. (2012) 48:4591–3. 10.1039/c2cc17766h 22473513

[68] Lu C Sun F Liu Y Xiao Y Qiu Y Mu H. Versatile Chlorin e6-based magnetic polydopamine nanoparticles for effectively capturing and killing MRSA. Carbohydr Polym. (2019) 218:289–98. 10.1016/j.carbpol.2019.05.007 31221332

[69] Fallows SJ Garland MJ Cassidy CM Tunney MM Singh TR Donnelly RF. Electrically-responsive anti-adherent hydrogels for photodynamic antimicrobial chemotherapy. J Photochem Photobiol B. (2012) 114:61–72. 10.1016/j.jphotobiol.2012.05.011 22677563

[70] Kirtane AR Verma M Karandikar P Furin J Langer R Traverso G. Nanotechnology approaches for global infectious diseases. Nat Nanotechnol. (2021) 16:369–84. 10.1038/s41565-021-00866-8 33753915

