# Supplementary material for: Stimuli-Responsive Nanoplatform-Assisted Photodynamic Therapy Against Bacterial Infections
Source: Front Med (Lausanne). 2021 Sep 13;8:729300. doi: 10.3389/fmed.2021.729300 (PMC8482315; doi:10.3389/fmed.2021.729300)
Supplement: Supplementary file 1 [file Table_1.docx]

**Supplementary Table 1. Clinical trials of PDT in fight against bacterial infections.**

National Clinical Trial (NCT) number is a unique number given by (ClinicalTrials.gov) to identify

a clinical trial registered on their website. Not applicable is used to describe trials without FDA-defined phases, including trials of devices or behavioral interventions. [the datasets for this table can be found in the (ClinicalTrials.gov) (<https://clinicaltrials.gov/>) with a search criterion of ((anti-infective OR antimicrobial OR antibacterial) AND photodynamic)].

| **NCT Number** | **Conditions** | **Bacteria Species** | **PS** | **Phases** |
| --- | --- | --- | --- | --- |
| NCT04857346 | Type 2 Diabetes Mellitus; Prediabetic State;  Chronic Periodontitis | *P. gingivalis* | Indocyanine Green | Phase 2\|Phase 3 |
| NCT04842188 | Furcation Defects | Not Mentioned | Not Mentioned | Not Applicable |
| NCT04587089 | Necrotic Pulp | Total Bacteria Infected | Methylene Blue | Phase 1\|Phase 2 |
| NCT04532060 | Health Care Associated Infection | Total Bacteria Infected | Photodithazine | Phase 2 |
| NCT04374383 | Periodontitis;  Alveolar Bone Loss | Total Bacteria Infected | Phthalocyanine | Phase 2 |
| NCT04187053 | Dent Implants;  Laser | Total Bacteria Infected | Methylene Blue | Phase 4 |
| NCT04047914 | Therapy, Photodynamic; Renal Insufficiency; Chronic | MRSA;  *S.aureus* | Methylene blue | Not Applicable |
| NCT03996044 | Halitosis | *P. gingivalis*;  *T. forsythia*;  *T. denticola* | Urucum | Not Applicable |
| NCT03904641 | Molar Incisor Hypomineralization | Total Bacteria Infected | Not Mentioned | Phase 1 |
| NCT03855345 | Periodontal Diseases | Total Bacteria Infected | Not Mentioned | Phase 2 |
| NCT03826810 | Molar Incisor Hypomineralization | Total Bacteria Infected | Not Mentioned | Phase 1 |
| NCT03818906 | Tooth Extraction Status Nos | Not Mentioned | Not Mentioned | Not Applicable |
| NCT03763110 | Pulp Necrosis | Not Mentioned | Not Mentioned | Not Applicable |
| NCT03757260 | Periodontitis | Total Bacteria Infected | Methylene Blue | Not Applicable |
| NCT03753958 | Peri-Implantitis | Total Bacteria Infected | Methylene Blue | Phase 2 |
| NCT03704857 | Endodontically Treated Teeth;  Postoperative Pain | Not Mentioned | Methylene Blue | Not Applicable |
| NCT03656419 | Halitosis | Microorganism related to halitosis | methylene blue | Not Applicable |
| NCT03576105 | Pericoronitis | *Tf* | methylene blue | Not Applicable |
| NCT03498404 | Chronic Periodontitis | 40 Subgingival Bacterial Species | Phenothiazine | Phase 4 |
| NCT03483376 | Dental Plaque;  Tooth Discoloration | Not Mentioned | Curcumin | Not Applicable |
| NCT03462368 | Gingival Recession; Generalized | Not Mentioned | Toluidine Blue O | Not Applicable |
| NCT03380403 | Diabetic Foot Infection; Osteomyelitis;  Photo Antimicrobial Chemotherapy | Not Mentioned | Methylene blue | Not Applicable |
| NCT03346460 | Halitosis | Total Bacteria Infected | Urucum | Phase 2 |
| NCT03309748 | Dental Deposits;  Tooth Discoloration;  Dental Prophylaxis | *A. actinomycetemcomitans*;  *T. forsythia*;  *P. gingivalis* | Not Mentioned | Not Applicable |
| NCT03308019 | Chronic Periodontitis; Smoking | Total Bacteria Infected | Methylene blue | Not Applicable |
| NCT03270254 | Periodontitis | Not Mentioned | Not Mentioned | Not Applicable |
| NCT03264118 | Periodontitis, Chronic; Periodontal Diseases; Periodontal Bone Loss | Not Mentioned | Toluidine Blue O | Not Applicable |
| NCT03222544 | Diabetic Extremity Lower Ulcers | Total Bacteria Infected | Methylene Blue | Not Applicable |
| NCT03212729 | Photochemotherapy Reaction;  Dental Pulp Necrosis; Polymerase Chain Reaction; Infection;  Enterococcal Infections; Candida | *E. faecalis*;  Others | Methylene Blue | Not Applicable |
| NCT03186352 | Primary Root Caries | *S. mutans*; *Lactobacillus spp*; *Actinomyces spp* | Hematoporphyrin | Not Applicable |
| NCT03142776 | Aggressive Periodontitis; Generalized | Not Mentioned | Not Mentioned | Not Applicable |
| NCT03140059 | Aggressive Periodontitis; Generalized | Not Mentioned | Not Mentioned | Not Applicable |
| NCT03132714 | Aggressive Periodontitis; Generalized | Not Mentioned | Not Mentioned | Phase 2\|Phase 3 |
| NCT03102892 | Diabetes Mellitus, Type 1; Periodontal Diseases; Periodontal Pocket | Not Mentioned | Methylene Blue | Not Applicable |
| NCT03095378 | Gingival Recession | Not Mentioned | Toluidine Blue O | Not Applicable |
| NCT03074136 | Teeth, Endodontically-Treated | Total Bacteria Infected | Phenothiazine Chloride | Phase 2 |
| NCT03039244 | Chronic Periodontitis | Total Bacteria Infected | Phenothiazine Hydrochloride | Not Applicable |
| NCT02938988 | Periodontal Diseases | Not Mentioned | Methylene Blue | Not Applicable |
| NCT02929927 | Root Canal Disinfection | Total Bacteria Infected | Not Mentioned | Not Applicable |
| NCT02929914 | Dental Caries | *S. mutans*;  *L. casei*;  Others | Toluidine Blue O | Phase 1 |
| NCT02908789 | Dental Caries | *S. mutans*;  Total Streptococcus; *Lactobacillus spp* | Methylene Blue | Not Applicable |
| NCT02848482 | Peri-implantitis | Total Bacteria Infected | Not Mentioned | Phase 3 |
| NCT02734784 | Chronic Periodontitis | 40 Subgingival Bacterial Species | Phenothiazine Hydrochloride | Phase 4 |
| NCT02627534 | Chronic Periodontitis;  Diabetes Mellitus | Not Mentioned | Methylene Blue | Not Applicable |
| NCT02555488 | Periradicular Disease | Total Bacteria Infected | Methylene Blue | Phase 2 |
| NCT02514226 | Bronchiectasis;  Periodontal Disease | Total Bacteria Infected | Methylene Blue | Not Applicable |
| NCT02479958 | Dental Caries | *S. mutans*;  *S. sobrinus*;  *L. casei*;  *F. nucleatum*;  *A. rimae*;  Others | Toluidine Blue O | Phase 1 |
| NCT02337192 | Dentist-Patient Relations | Total Bacteria Infected | Curcumin | Phase 1 |
| NCT02075671 | Rosacea;  Papulopustular Rosacea | Not Mentioned | 5-Aminolevulinic Acid | Phase 4 |
| NCT02049008 | Aggressive Periodontitis | 40 Subgingival Bacterial Species | Phenothiazine Hydrochloride | Phase 4 |
| NCT02043340 | Periodontitis | Total Bacteria Infected | Indocyanine Green | Phase 2 |
| NCT01964833 | Periodontitis;  Diabetes Mellitus Type 2 | Not Mentioned | Methylene Blue | Not Applicable |
| NCT01854619 | Chronic Sinusitis | Total Bacteria Infected | Methylene Blue | Not Applicable |
| NCT01595594 | Periodontal Disease;  Type 2 Diabetes | Total Bacteria Infected | Not Mentioned | Phase 3 |
| NCT01532674 | Chronic Periodontitis | Not Mentioned | Methylene Blue | Phase 4 |
| NCT01245946 | Acne | Not Mentioned | 5-Aminolevulinic Acid | Phase 2 |
| NCT00825760 | Chronic Leg Ulcers; Wound Healing | Anaerobes;  MSSA;  MRSA; *P.aeruginosa*;  *BHS* | PPA904 | Phase 2 |

Abbreviations: *P. gingivalis*, *Porphyromonas gingivalis*; MRSA, Methicillin-resistant *Staphylococcus aureus*; *S. epidermidis*, *Staphylococcus epidermidis*; *T. forsythia*, *Tannerella forsythia*; *T. denticola, Treponema denticola*；*Tf*, *Tannarella forsythia*; *A. actinomycetemcomitans*, *Aggregatibacter actinomycetemcomitans*; *T. forsythia*, *Tannerella forsythia*; *E. faecalis*, *Enterococcus faecalis*; *S. mutans, Streptococcus mutans*; *L. casei, Lactobacillus casei*; *S. sobrinus*, *Streptococcus sobrinus*; *F. nucleatum*, *Fusobacterium nucleatum*; MSSA, Methicillin-sensitive *Staphylococcus aureus*; MRSA, Methicillin-resistant *Staphylococcus aureus*; *P. aeruginosa*, *Pseudomonas aeruginosa*; *BHS*, *beta-haemolytic Streptococci*
